# Significance of Selected Environmental and Biological Factors on the Risk of FASD in Women Who Drink Alcohol during Pregnancy

**DOI:** 10.3390/jcm12196185

**Published:** 2023-09-25

**Authors:** Elżbieta Grzywacz, Bogusław Brzuchalski, Małgorzata Śmiarowska, Damian Malinowski, Anna Machoy-Mokrzyńska, Monika Anna Białecka

**Affiliations:** 1 Department of Pharmacokinetics and Therapeutic Drug Monitoring, Pomeranian Medical University, Aleja Powstanców Wielkopolskich 72 St., 70-111 Szczecin, Poland; elagrzywacz@me.com (E.G.); machalin@googlemail.com (B.B.); malgorzata.smiarowska@pum.edu.pl (M.Ś.); monika-bialecka@post.pl (M.A.B.); 2 Department of Experimental and Clinical Pharmacology, Pomeranian Medical University, Aleja Powstanców Wielkopolskich 72 St., 70-111 Szczecin, Poland; anna.machoy@pum.edu.pl

**Keywords:** FASD, pregnancy, prenatal alcohol exposure

## Abstract

Prenatal alcohol exposure (PAE), which refers to alcohol consumption by pregnant women, is associated with the risk of numerous severe complications during fetal development. The State Agency for Alcohol Problem Solving reports that the incidence of fetal alcohol spectrum disorder (FASD) in Poland’s general population is over 1.7%, and the incidence of fetal alcohol syndrome (FAS) is estimated at more than 0.5%. This study aimed to evaluate the significance of alcohol exposure and focused on the pattern of alcohol intoxication exhibited by the mother during pregnancy and other environmental factors of the maternal environment contributing to the development of FASD. The study covered 554 subjects, including 251 mothers and 303 children (213 girls and 90 boys). The mother’s drinking problem was determined based on the information obtained from the case history. All children qualified for the study fulfilled the h-PAE (high alcohol exposure) criteria during their fetal life. The clinical diagnosis of FAS and pFAS (occurrence of morphological symptoms of fetal alcohol syndrome) was made using a four-digit diagnostic questionnaire validated in the Polish version of the Washington Questionnaire for the assessment of the spectrum of alcohol-related neurodevelopmental disorders or alcohol-related cognitive impairment (ARND/C). Statistical analysis of the obtained research results was developed using statistical software–STATISTICA PL, version 13.1 (StatSoft, Inc., Szczecin, Poland 2016, STATISTICA–data analysis software system, version 13.1). The most destructive drinking behaviors are compulsive intoxication (BD, binge drinking) during the first 6 weeks of pregnancy and chronic addiction throughout its duration (CHD, chronic drinking). Chronic alcohol intoxication (CHD) leads to a poorer nutritional status in mothers, which is reflected in a lower body mass index (BMI) (<18 kg/m^2^).

## 1. Introduction

Prenatal alcohol exposure (PAE), which refers to alcohol consumption by pregnant women, is associated with the risk of numerous severe complications during fetal development. The effects of PAE are conceptualized as fetal alcohol spectrum disorder (FASD). The criteria for fetal alcohol spectrum disorder (FASD) are met by 30–40% of children with significant prenatal alcohol exposure, known as high prenatal alcohol exposure (h-PAE). The prevalence of FASD in 76 countries is more than 1% and is high in individuals living in out-of-home care or who are engaged with justice and mental health systems [1]. The State Agency for Alcohol Problem Solving reports that the incidence of fetal alcohol spectrum disorder (FASD) in Poland’s general population is over 1.7%, and the incidence of fetal alcohol syndrome (FAS) is estimated at more than 0.5%. However, these results may be underestimated [2]. Fetal alcohol spectrum disorder (FASD) is a broader diagnosis that includes patients with fetal alcohol syndrome (FAS), as well as those who have been affected by prenatal alcohol exposure but fail to meet the complete criteria for FAS [3]. Children exposed to prenatal alcohol intoxication may exhibit full-blown fetal alcohol syndrome (FAS). A diagnosis of FAS is based on the clinical features of physical disabilities, including morphological prenatal and/or postnatal growth retardation, facial dysmorphology, and central nervous system dysfunction, as well as neurobehavioral disabilities, which need to be present [3]. A minimum of two morphological changes indicating FASD should be present in partial fetal alcohol syndrome (pFAS). Morphological changes observed in FASD include smaller head dimensions, lower height, and lower body mass index (BMI) [4]. Advances have been made in diagnosing and treating fetal alcohol spectrum disorders [5], disorders within the skeletal system, kidneys, hearing organs, and heart [6]. The most characteristic dysmorphic changes are facial and include flat nasal epiphysis, flat philtrum, short and upturned nose, and thin upper lip [7].

The FASD spectrum includes syndromes such as alcohol-related birth defects (ARBD), alcohol-related neurodevelopmental disorders (ARND), and neurobehavioral disorders associated with prenatal alcohol exposure (ND-PAE) [8,9]. The Diagnostic and Statistical Manual of Mental Disorders–Fifth Edition (American Psychiatric Association, 2013) recently included ND-PAE in its ‘conditions for further study’ section. ND-PAE is based on the diagnostic DSM-V criteria, which require a documented history of prenatal alcohol exposure identifying specific areas of neurobehavioral deficits, self-regulation, and adaptive functioning domains. The proposed diagnosis can manifest irrespective of physical dysmorphology related to prenatal alcohol exposure, and both FAS/PFAS and ND-PAE diagnoses can be provided [9]. 

Very significant consequences of h-PAE within the FASD spectrum relate to the central nervous system with regard to cognitive decline. They have a more severe developmental dimension and are not phenotypically evident. Approximately two thirds of children diagnosed with this condition may experience a lower IQ, difficulties in logical thinking, attention deficits, and behavioral and emotional disorders, which can cause reluctance to learn and significant deterioration in school functioning, as well as lower cognitive development, especially in the area of intellectual ability, memory, attention, and speech [10,11]. This is particularly relevant to children diagnosed with alcohol-related neurodevelopmental disorder or alcohol-related cognitive impairment (ARND/C). As a result, determining exposure’s behavioral and mental effects on the central nervous system without physical indications, such as characteristic facial features and growth deficiency, can prove challenging for many [12].

Insufficient evidence is available to determine the extent to which alcohol consumption should be taken into account in relation to the risk of FASD, as the safe dose of alcohol use during pregnancy has never been established [13,14]. 

A possible factor that influences the development of FASD is the pattern of alcohol consumption during pregnancy, which can result in varying degrees of the fetus’s exposure to alcohol. It is highly harmful (toxic) for the development of the embryo or fetus when alcohol is consumed by the mother during the so-called high prenatal alcohol exposure (h-PAE) [15,16], for which sinusoidal or linear models are distinguished [17,18,19,20]. Another classification of alcohol intoxication in pregnant women is based on the dynamics of alcohol consumption, i.e., the quantity of alcohol consumed at a particular time. It includes chronic alcohol consumption when individuals consume more than four standard units of pure ethanol (>40 mL/d) at least twice a week, amounting to no less than eight times per month (>320 mL/month) [21]. Alcohol abuse leading to dipsomania refers to consuming over 80 mL of pure ethanol at least once a week, which amounts to more than 320 mL per month. During pregnancy, incidental drunkenness is defined as significantly pathological if the state of drunkenness is a result of consuming a single dose of more than 100 mL, without the need for repeating doses. In the case of pregnant women, all the patterns of drinking involve high prenatal alcohol exposure (h-PAE) [21] due to the toxic concentration of ethyl alcohol (Table 1), which is highly harmful to the development of the embryo or fetus [22].

However, it is essential to note protective factors when examining the long-term effects of h-PAE. These include the appropriate amount and proportion of nutrients necessary for the proper functioning of the mother’s body and, consequently, the development of the fetus. The number of kilocalories provided in the diet and the mother’s lifelong and current nutrition (body weight, fat content, and BMI) have a substantial impact on the relative risk for FASD [23,24] including its more severe FAS syndrome [4,25].

Alcohol pharmacokinetics is closely linked to adipose tissue. When the fat content is lower, and, subsequently, the BMI of the mother is also lower, the concentration of alcohol in the blood of both the mother and the fetus increases, which leads to a greater amount of alcohol absorbed into the developing fetus’s brain due to the higher fat content in the central nervous system. This, however, exacerbates organic damage in children exposed to h-PAE [26].

Within 120 min of the mother’s consumption of alcohol, the level of blood alcohol content, BAC, rises to its maximum. Due to transplacental transport, the level of alcohol in the fetus is nearly the same as that of the mother. Additionally, after 120 min, it reaches the highest BAC value obtained in the mother [21].

One of the effects of h-PAE is a decrease in protein expression related to the transplacental transport of folic acid, resulting in impaired elimination of formaldehyde. This compound is neurotoxic and formed from methanol when reactive oxidative species (ROS) are in excess [27]. Increased ethanol consumption by pregnant women leads to higher methanol production, thereby intensifying oxidative stress, i.e., the formation of by-products of oxidative phosphorylation in the mitochondria. The rise in formaldehyde production further increases the amount of reactive oxidative species. When formaldehyde elimination is reduced, the level of formaldehyde quickly increases, leading to a vicious cycle mechanism. The increase disrupts the elimination processes, which, in turn, increases the number of substrates for formaldehyde formation [28].

Furthermore, evidence indicates that individuals who consume more than 30% of their total caloric intake as alcohol often consume insufficient amounts of daily recommended nutrients, including carbohydrates, proteins, fats, vitamins, and minerals such as calcium and iron. Pregnant women often fail to consume the recommended amounts of micronutrients, which include some that may be crucial in FASD, such as choline, folate, vitamin B12, iron, and vitamin A [4]. In the United Kingdom, a study of women who abused alcohol—both during pregnancy and outside of it—discovered that alcohol consumption can exacerbate micronutrient deficiencies (such as selenium, calcium, and zinc) and, in particular, deficiencies in vitamins A, E, B1, B2, B6, C, D, and folate due to the reduction in antioxidant levels and the increase in toxic metabolites, including acetaldehyde. Thiamine is a cofactor for dehydrogenase enzymes, while pyridoxine is a cofactor for decarboxylase enzymes, both of which are necessary for oxidative glucose metabolism. Previous studies have suggested that women who engage in risky alcohol consumption tend to choose alcohol over nutrient-dense foods, leading to hypoglycemia and micronutrient deficiency [29]. The function of vitamins and micronutrients as cofactors for enzymes in protein and carbohydrate metabolism, e.g., in the Krebs cycle, may account for the clinical effects of h-PAE, including oxidative stress, cellular apoptosis, and micro-encephalopathy. Insufficient maternal caloric intake, low pre-pregnancy weight and inadequate weight gain during pregnancy are additional risk factors for intrauterine growth retardation (IUGR), irrespective of alcohol consumption. Furthermore, malnourished individuals with alcohol addiction metabolize alcohol at a slower pace, which results in elevated levels of alcohol in the blood [4].

## 2. Materials and Methods

### 2.1. Study Group

The study covered 554 subjects, i.e., 251 mothers aged 32.77 ± 5.93 and 303 children—213 girls aged 8.59 ± 4.77 and 90 boys aged 9.77 ± 5.14. The women were recruited in the province of West Pomerania, Poland. The drinking problem of the mother was determined on the basis of the information obtained from the case history. The manner of alcohol intoxication was considered and verified by documented social history, including the mother’s stay in the drunk tank and/or detoxification ward during pregnancy (Table 1). Mothers were interviewed regarding their social history, smoking behavior, and use of psychoactive substances (excluding alcohol), such as marijuana, amphetamine, cocaine, heroin, sleeping pills, and sedatives. Polytoxicomania was excluded from all the mothers examined in the study. The study covered only children with h-PAE whose mothers failed to meet the criteria for addiction or harmful use of psychoactive substances, excluding alcohol and nicotine. The group of females examined in the study exhibited a homogeneous pattern of alcohol intoxication. Females with chronic and debilitating diseases and endocrine disorders that may affect nutritional status and body weight (decompensated thyroid disorders, diabetes, and adrenal diseases) were excluded from the study. Intellectual ability was evaluated through an interview about their educational background and work experience. Additionally, it was verified by a psychiatric examination which determined the pattern of alcohol intoxication exhibited by the mother.

All children eligible for the study met the criteria for high alcohol exposure (h-PAE) during fetal development. Out of 303 children with h-PAE, 183 children with present phenotypic (morphological) FASD-related changes (All FAS Children, AFC) qualified for additional studies. Full-blown FAS was confirmed in 141 children, while 42 were diagnosed with partial pFAS. The control group consisted of 120 children with h-PAE, who displayed no morphological traits of FASD. They are referred to as non-FAS Children (NFC), as shown in Table 2.

The clinical diagnosis of FAS and pFAS (occurrence of morphological symptoms of fetal alcohol syndrome) was established using a four-digit diagnostic questionnaire validated in the Polish version of the Washington Questionnaire for the assessment of the spectrum of alcohol-related neurodevelopmental disorders or alcohol-related cognitive impairment (ARND/C). In order to measure phenotypic features of the face, computer analysis of digital facial photographs using FAS FACIAL PHOTOGRAPHIC Analysis Software Version 2.1 which analyses facial dysmorphic features that are typical of FAS for the Caucasian race, was used in the study [30]. 

The exclusion criteria for h-PAE children comprised psychiatric disorders, including psychotic (present and in medical history), significant mood and anxiety disorders that require pharmacological treatment, psychoactive substances (SPA) addiction, risky and harmful use of SPA (present and in medical history), as well as developmental disorders, including autism, profound and significant mental retardation, genetic diseases, severe and uncompensated somatic diseases (endocrinological, cardiovascular, renal, neoplastic, autoimmune, anorexia), as well as organic diseases with clinical manifestation (epilepsy). A minor deficiency in weight and height, but still within normal limits (>10th centile), as well as a history of a depressive episode and emotional and behavioral disorders did not result in the exclusion from the study.

The legal guardians/mothers of all children examined in the study had granted their written consent prior to the study. Written consent was also obtained from all children over the age of 16 [31]. The study was approved by the local Bioethics Committee at the Pomeranian Medical University in Szczecin (the approval of the Bioethics Committee of the Pomeranian Medical University in Szczecin from 5 October 2015, no. KB-0012/95/15). The project was financed by the Minister of Science and Higher Education program in Poland under the name “Regional Initiative of Excellence” in 2019–2022, project number 002/RID/2018/19.

### 2.2. Statistical Analysis

Statistical analysis of the research results was made using statistical software–STATISTICA PL, version 13.1 (StatSoft, Inc. 2016, STATISTICA–data analysis software system, version 13.1, www.statsoft.com accessed on 5 April 2019). The chi-squared test (χ2-Pearson’s test) and/or Fisher’s exact test were used to compare the frequencies of alleles and genotypes between the groups and to examine associations between clinical features and genotypes. The non-parametric Kruskal–Wallis test was used to analyze the significance of particular genotypes in relation to the age of subjects qualified for the study. Additionally, we verified if the frequencies of the observed alleles and genotypes conformed to Hardy–Weinberg’s law predictions. The values of analysis were statistically significant at a significance level of *p* < 0.05.

## 3. Results

The drinking patterns of all women in the study met the criteria for h-PAE in the fetus. Clinical interviews were conducted among 251 mothers of children with h-PAE to assess their alcohol consumption patterns. It was found that 98 out of 251 women (39% of mothers of children with h-PAE) had a high level of chronic alcohol exposure (CHD), which indicated their absolute alcohol addiction (AAA). An extreme drinking pattern indicating alcohol addiction or harmful drinking, referred to as alcohol abuse (ALAB), was exhibited in 19% of the study group, i.e., 48 women, in which control over drinking is illusory. In 20% of the study group, i.e., 51 women, ethanol incidentally was consumed in the so-called “party style” referred to as weekend binge drinking (WBD). Although they showed no signs of addiction, this behavior resulted in significant health damage (ARD), even when the total amount consumed over time was less than that of moderate drinkers. They required the implementation of preventive procedures related to alcoholism.

Of all the mothers in the study, 54 (22% of the study group) consumed ethanol by exhibiting the early binge drinking (EBD) pattern. This group comprised the youngest women under the influence of high levels of alcohol until the 6th week of pregnancy because they were unaware of possible health consequences.

Most children with h-PAE were born to mothers who were chronic drinkers (1.34 and 1.3 children per mother), whereas those who drank alcohol ignorantly during the first six weeks of pregnancy had the least number of children (1.06 and 1.04 children per mother).

Table 1 presents detailed characteristics of the mothers’ alcohol intoxication.

The age of mothers with children affected by h-PAE and showing morphological symptoms of FASD was 32.8 (SD = 5.93). In the FAS group, the mother’s average age was 32.9 (SD = 6.13), whereas, in the pFAS group, the average age was 30.9 (SD = 5.21). Mothers from the NFC group were at an average age of 34.2 (SD = 4.34)—as shown in Table 2.

No association was found in the study population between maternal age and the risk of FASD in children with h-PAE (FC/NFC–*p* = 0.8262).

Mothers of children exposed to h-PAE with FAS/pFAS symptoms (m-AFC group) had a significantly lower BMI (*p* < 0.0001). They were characterized by lower intellectual ability (*p* = 0.0059) compared with mothers of children without FAS/pFAS symptoms (m-NFC group). Mothers of children without symptoms of FAS/pFAS (NFC) showed features of being overweight (BMI > 25 kg/m^2^) and mothers of children with symptoms of FAS/pFAS (AFC) showed features of being underweight and malnourished (BMI < 18 kg/m^2^). Mothers of children exhibiting FAS/pFAS (m-AFC) symptoms were more likely to smoke during pregnancy and experienced fewer somatic diseases. However, these differences were not statistically significant compared with mothers of children without FAS/pFAS (m-NFC) symptoms. The study revealed no chronic diseases in the group of mothers, which would significantly affect the nutritional status of the children in the study concurrently with the metabolic effects associated with alcohol intoxication (Table 3).

Children diagnosed with FAS (AFC group, *n* = 98) were the majority in the group of mothers with CHD (chronic high alcohol drinking), i.e., mothers who drank alcohol excessively throughout pregnancy, with severe alcohol dependence, and high and systematic exposure to h-PAE.

The majority of children affected by h-PAE, 131 out of 303, were born to mothers who were chronic alcohol drinkers (CHD)—98 out of 251 mothers. The group of mothers, who were chronic alcohol drinkers (CHD), included the highest number of children with FASD-related changes and full-blown fetal alcohol syndrome (FAS)—82 out of 141 children. The incidence of FASD-related morphological changes with the clinical manifestation of FAS in children is similar in the groups of mothers exhibiting the drinking pattern which indicates addiction or harmful drinking (ALAB) and compulsive drinking (WBD and EBD)—12, 17, and 22 out of 141 children, respectively.

In the group of mothers exhibiting the ALAB and EBD patterns, the frequency of full-blown FAS syndrome is significantly higher than that of pFAS (20 out of 8 children and 22 out of 10 children, respectively). Conversely, in the WBD group, the incidence of FAS and pFAS is comparable (17 out of 15 children). Table 4 presents exposure to h-PAE, depending on the pattern of alcohol intoxication and the occurrence of morphological changes associated with the incidence of fetal alcohol syndrome.

In all the groups of children with h-PAE, girls were the majority in the FAS, pFAS, and NFC groups compared with boys, respectively: 60% (*n* = 84, aged 8.32 ± 3.92 vs. *n* = 57, aged 9.75 ± 4.48), 88% (*n* = 37, aged 8.58 ± 4.51 vs. *n* = 5, aged 10.6 ± 3.13), and 76% (*n* = 92, aged 8.43 ± 4.39 vs. *n* = 28, aged 9.64 ± 6.62) (Table 4).

## 4. Discussion

The results suggest that the drinking pattern exhibited by pregnant women is significant for both the development of FASD-related organic changes and fetal development. The study confirms that the symptoms of damages caused by fetal alcohol spectrum disorder (FASD) are manifested through morphological changes in both full-blown fetal alcohol syndrome (FAS) and partial pFAS (AFC group, *n* = 98). They are most frequently found in children whose mothers drink alcohol excessively throughout pregnancy (CHD, chronic high alcohol drinking), with deep alcohol dependence and high and systematic exposure to h-PAE. This was found in 43.23% of children in the study (chi2 = 32.3087, *p* = 0.000014) (Table 4). Furthermore, it was found that women who gave birth to children with FAS/pFAS symptoms (m-AFC) had a lower BMI than mothers of children without these symptoms (*p* < 0.0001), which may, on the one hand, indicate greater neglect of women in terms of nutritional supply (excluding chronic diseases affecting nutritional status), and, on the other hand, reflect greater fetal exposure to the neurotoxic effects of alcohol when lower adipose tissue resources are available.

Mothers of children with FAS/pFAS (m-AFC) symptoms were more likely to be underweight than mothers of children without FAS/pFAS (NFC) symptoms. Using alcohol during pregnancy can harm the child’s development, especially if the woman is underweight. Our study has also found that children of underweight mothers, who drank alcohol, have a higher chance of being born with fetal alcohol syndrome (FAS). Carter et al. [32] found that children of mothers with lower prepregnancy weight may experience greater reductions in weight, height, BMI, and head circumference due to alcohol consumption. Such effects may persist into adulthood. This could be because smaller mothers tend to have higher blood alcohol concentrations when consuming alcohol [4]. Previous studies have demonstrated that alcohol and drug consumption can alter nutrient absorption, impairing the quality and quantity of nutrient and energy intake and thus inducing malnutrition, particularly of micronutrients such as vitamins, omega-3, folic acid, zinc, choline, iron, copper, and selenium [1]. Inadequate nutrient intake, particularly of riboflavin, calcium, and zinc, also increases the risk of fetal alcohol syndrome (FAS) [5]. Maternal consumption of alcohol and drug abuse may compromise the nutrition available for the fetus, leading to a shortage of essential nutrients and resulting in fetal abnormalities such as intrauterine growth restriction or fetal alcohol spectrum disorder [1]. A smaller body pattern (indicated by height, weight, and body mass index) is also associated with an increased prevalence of FAS [3]. It should be noted that comparing relevant research results from different countries may lead to imprecise interpretations due to variations in research methodology. Therefore, it is necessary to conduct multicenter international research with a standardized methodology that makes it possible to compare the results [3]. The results are consistent with other authors’ observations that mothers of children with FAS have a low BMI. Additionally, it is more probable that smaller women have a lower average of alcohol consumption, resulting in FAS symptoms, compared with larger women [4]. Previous studies have indicated that inadequate maternal nutrition in women who consume alcohol harmfully during pregnancy is associated with higher rates of fetal alcohol spectrum disorder (FASD) [2,32,32,33] found that fetal alcohol spectrum disorder (FASD) is more likely to develop in women with a lower body mass index (BMI) and higher alcohol concentration in the blood. However, maternal weight gain during pregnancy did not alleviate the effects of alcohol consumption on the development of fetal alcohol spectrum disorder (FASD) [25]. The results indicate that not only the manner in which the fetus consumes alcohol but also the duration of exposure to intoxication affects the occurrence of damage related to FASD and the severity of its clinical manifestations. The neurotoxic effect of alcohol on the fetal CNS is closely related to its pharmacokinetics. One crucial aspect is that the level of alcohol concentration in the mother’s blood rises to its maximum (blood alcohol content, BAC) within 120 min after ingestion. At the same time, as a result of transplacental transport, the fetal alcohol level is almost identical to the maternal level, and approaches the maternal peak BAC after 120 min [34].

Our research study found that the occurrence of FASD-related morphological changes was similar in the groups of mothers who engaged in compulsive drinking (WBD, weekend binge drinking) and early binge drinking (EBD). However, in the EBD group, which experienced exposure to h-PAE in the first six weeks of pregnancy, the incidence of full-blown FAS was significantly higher than in the WBD group. In the WBD group, the incidence of both full-blown FAS and partial pFAS was approximately the same (Table 4). The above study confirms the well-known and accepted thesis that any amount of alcohol consumed during pregnancy is toxic to the fetus. Under h-PAE conditions, the early stage of alcohol exposure is crucial, particularly when the mother is unaware that she is pregnant (EBD). Early alcohol exposure increases the risk factor for the onset and severity of fetal alcohol spectrum disorder (FASD). Even if drinking is controlled later in pregnancy and similar to earlier levels, it is widely recognized as highly harmful. Previous studies have indicated that women who consume alcohol, especially in large quantities, have an increased risk of morbidity and mortality in their newborns [7,32,33,35].

In our study, the difference between mothers that smoked and intoxicated themselves with alcohol in a harmful manner, who gave birth to children with FAS/pFAS symptoms, and mothers of children without clinical symptoms of FAS/pFAS was not statistically significant (*p* > 0.05, Table 4). It can be assumed that smoking by the women in the study did not increase the risk of FAS/pFAS in the group of children with h-PAE. However, it cannot be ruled out that chronic alcohol drinking (CHD) by pregnant women, combined with their increased smoking, may have had an impact on their dietary habits, resulting in a lower BMI in mothers of children with FAS/pFAS (m-AFC) symptoms (Table 4). The relationship between cigarette smoking and alcohol consumption was also highlighted by other authors, who showed that cigarette smoking rates during pregnancy are often higher in women who consume alcohol. This fact may be related to the mother’s nutritional status and may indirectly limit the child’s growth, cause its low birth weight, and increase the risk of preterm birth [36,37,38,39,40,41]. In addition, tobacco smoking can increase carboxyhemoglobin levels, which can impair fetal oxygenation and fetal growth [6]. Determining the association between cigarette smoking by women who are intoxicated and the risk of FASD requires further prospective research, as it may predispose to developmental disorders, including cognitive disorders that manifest later in the development, e.g., in the group of ARNDs (alcohol-related neurodevelopmental disorders), as well as neurobehavioral disorders associated with fetal alcohol exposure—ND-PAE (neurobehavioral disorders associated with prenatal alcohol exposure). ADHD has been shown to be a major complication of PAE, especially if the mother smoked during pregnancy.

The available publications emphasize that mothers, who gave birth to children with FASD, had higher rates of substance use disorders, personality disorders, mood disorders, and anxiety disorders prior to pregnancy with the child. The most common substances of abuse used during pregnancy are cocaine, methamphetamine, and cannabinoids, resulting in multiple nutrient deficiencies and malnutrition [6]. Maternal exposure to cannabinoids during pregnancy affects fetal brain development [36,37,38], preterm birth, low birth weight [6,38], and subsequent behavioral self-regulation [32,35]. Methamphetamine may deregulate neurotransmitter circulation and cause adverse effects on the fetus, especially since the use of methamphetamine among young pregnant patients appears to be increasing [39]. Pregnant women who used cocaine weighed significantly less before pregnancy and gained slightly less weight during pregnancy [40]. In addition, prenatal cocaine exposure has been associated with poor maternal weight gain, IUGR, placental abruption, preterm and abrupt labour, fetal distress, various birth defects and neonatal neurobehavioral deficits, low birth weight, and small cranial circumference [41]. Pregnant women who used cocaine or cannabis had low ferritin and folate levels, influencing nutrition [42]. Maternal heroin use is associated with IUGR, preterm delivery, and increased neonatal mortality. Some women who use heroin also use other harmful substances—tobacco, alcohol, and cocaine [6]. Taking into account the above observations, the effects of psychoactive substances other than alcohol on the increased risk of FASD when the fetus was exposed to h-PAE were not found in our study because none of the women used psychoactive substances other than alcohol in a harmful way, nor were they addicted to them (in the interview, only a few women reported occasional contact with other psychoactive substances). This is a limitation of the present study because observations of a larger group of patients, including mothers of children with h-PAE and polytoxicomania, could determine their significance for developing organic changes in their children, including the spectrum of FASD disorders.

The factor analyzed in the present research project was also the intellectual ability of the women who participated in the study. Children with FASD and a diagnosis of FAS/pFAS (AFC) were more likely to have mothers diagnosed with mental disabilities compared with children in the NFC group whose mothers had no intellectual disability. The risk of FASD-related central damage and clinical effects in children of mothers with mental disabilities and h-PAE alcohol intoxication is believed to be higher. This is partly due to choline deficiency in the diet of this group of women [23,43]. Because an undernourished mother has a limited supply of nutrients for the fetus, low postnatal nutrition can exacerbate the effects of malnutrition, resulting in growth retardation and impaired cognitive functions such as IQ and language development [6]. Therefore, the administration of choline early in pregnancy should be a preventive measure [23,43]. As a result, heavy prenatal alcohol exposure’s adverse effects on postnatal growth and cognitive functions, including non-verbal intelligence, visual–spatial skills, working memory ability, verbal memory, and fewer behavioral symptoms of attention deficit hyperactivity in human infants, may be mitigated [23,43]. Previous studies have shown that parents of children diagnosed with developmental disorders are more likely to experience mental health issues than parents of children without such symptoms [44]. Few studies have investigated the pregnancy course and delivery among women with intellectual disability and their infants when compared with other disabilities [15,16,45,46]. Research has shown that women with intellectual disabilities of reproductive age experience significant socioeconomic status and health disparities and are more likely to have chronic health conditions and mental illnesses [39,47,48,49]. This is why some women may be inclined to seek solutions for their functional and emotional problems through excessive use of alcohol or other substances, regardless of the consequences suffered by them and their children. This group is at a greater risk for unplanned pregnancies due to lower self-awareness and insufficient consideration of the potential consequences of their actions. Pregnancy may occur while consuming psychoactive substances, which often result in children being born with symptoms of fetal alcohol syndrome (FAS) or partial fetal alcohol syndrome (pFAS). These women, particularly in the absence of adequate environmental support, are less likely to initiate or sustain conscious abstinence.

## 5. Conclusions

According to our study, there is no safe pattern of alcohol consumption during pregnancy. Alcohol consumption increases the risk of developing fetal alcohol spectrum disorder (FASD). The most destructive drinking behaviors are compulsive intoxication (BD, binge drinking) during the first 6 weeks of pregnancy and chronic addiction throughout its duration (CHD, chronic drinking). Chronic alcohol intoxication (CHD) leads to a poorer nutritional status in mothers, which is reflected in a lower body mass index (BMI) (<18 kg/m^2^). Smoking and drinking during pregnancy can also impact the woman’s nutrition. However, this correlation is complicated and further research is required. Mothers of children with FASD have also been found to exhibit diminished intellectual abilities.

## Figures and Tables

**Table 1:** Characteristics of drinking patterns in mothers of children with h-PAE.

Ethanol Drinking Pattern	CHD	ALAB	WBD	EBD
Number of women in the study, *n* = 251 (100%)	*n* = 98 (39%)	*n* = 48 (19%)	*n* = 51 (20%)	*n* = 54 (22%)
Amount of ethanol [g]/occasion [time]	≥80 g/week	40–60 g	1× ≥ 100 g	≥80 g/120 min
Frequency [intoxication/week]	≥2×/week	≥2×/week ≤5×/week	1–4×/4 weeks	≥1×/4 weeks, maximum 4×/week
Duration of consumption during pregnancy	entire period	entire period	entire period	only until pregnancy was confirmed, ≥4 Hbd ≤6 Hbd
Number of children per mother [mean]	1.34	1.3	1.06	1.04

CHD—chronic drinking; ALAB—alcohol abuse; WBD—weekend binge drinking; EBD—early binge drinking.

**Table 2:** Demographic data of patients (mothers and children) in the study.

hPAE Children Group	*n*	Age [Years]	Female Sex [*n*]	Female Sex [%]
total	303	8.94 ± 4.90	213	70.30%
FC	141	8.96 ± 5.07	84	59.57%
PFC	42	8.60 ± 3.87	37	88.07%
NFC	120	9.03 ± 5.06	92	76.67%
Mothers Group	*n*	Age [Years]		
total	251	32.77 ± 5.93		
total (hPAE groups) *				
FC	141	33.38 ± 5.94		
PFC	42	33.14 ± 4.48		
NFC	120	32.59 ± 5.90		

hPAE—high prenatal alcohol exposure, FAS children—FC, partial FAS children—PFC, non-morphological FAS children—NFC; * counting mothers with more than one child.

**Table 3:** Characteristics regarding nutritional status, smoking, chronic diseases, and level of intellectual ability of mothers whose children were exposed to h-PAE during pregnancy.

Mothers of Children with h-PAE, *n* = 251	m-AFC	m-NFC	*p*
Cigarettes	72%	68%	*p* = 0.4564
BMI > 25	36%	62%	*p* < 0.0001
BMI < 18	5%	3%	*p* = 0.3972
Chronic diseases	8%	10%	*p* = 0.5483
Mother’s mental retardation	14%	3%	*p* = 0.0059

m-AFC—group of mothers whose children were diagnosed with FAS and pFAS, m-NFC—group of mothers whose children showed no FASD morphological symptoms (non-FAS children).

**Table 4:** The incidence of FAS and pFAS phenotypes in relation to the fetus’s exposure to h-PAE under various patterns of maternal alcohol intoxication.

Mothers of Children with h-PAE, *n* = 251	CHD	ALAB	WBD	EBD
FAS (*n* = 141)	82 [58.16%]	20 [14.18%]	17 [12.06%]	22 [15.60%]
pFAS (*n* = 42)	9 [21.43%]	8 [19.05%]	15 [35.71%]	10 [23.81%]
NFC (*n* = 120)	40 [33.33%]	34 [28.33%]	23 [19.17%]	23 [19.17%]
children with h-PAE, *n* = 303	131(43.23%)	62 (20.46%)	54 (17.49%)	56 (18.82%)
chi2 = 32.3087, *p* = 0.000014				

h-PAE—children exposed to high fetal alcohol intoxication, FAS—children with h-PAE and full-blown FAS syndrome, pFAS—children with h-PAE and partial FAS syndrome, NFC—a group of children without symptoms of FAS/pFAS (without morphological symptoms of FASD), CHD—chronic high alcohol drinking, ALAB—actual extent of alcohol abuse, WBD—weekend binge drinking, EBD—early binge drinking.

## Data Availability

Not applicable.
